# Future Projection of Extreme Precipitation Indices over the Qilian Mountains under Global Warming

**DOI:** 10.3390/ijerph20064961

**Published:** 2023-03-11

**Authors:** Yanzhao Li, Xiang Qin, Zizhen Jin, Yushuo Liu

**Affiliations:** 1 Northwest Institute of Eco-Environment and Resources, Chinese Academy of Sciences, Lanzhou 730000, China; 2 University of Chinese Academy of Sciences, Beijing 100049, China; 3 Qilianshan Observation and Research Station of Cryosphere and Ecologic Environment, State Key Laboratory of Cryospheric Science, Northwest Institute of Eco-Environment and Resources, Chinese Academy of Sciences, Lanzhou 730000, China

**Keywords:** extreme precipitation, future projection, CMIP6 model, bias correction, accuracy evaluation, Qilian Mountains

## Abstract

The Qilian Mountains are a climate-sensitive area in northwest China, and extreme precipitation events have an important impact on its ecological environment. Therefore, considering the global warming scenario, it is highly important to project the extreme precipitation indices over the Qilian Mountains in the future. This study is based on three CMIP6 models (CESM2, EC-Earth3, and KACE-1-0-G). A bias correction algorithm (QDM) was used to correct the precipitation outputs of the models. The eight extreme precipitation indices over the Qilian Mountains during the historical period and in the future were calculated using meteorological software (ClimPACT2), and the performance of the CMIP6 models to simulate the extreme precipitation indices of the Qilian Mountains in the historical period was evaluated. Results revealed that: (1) The corrected CMIP6 models could simulate the changes in extreme precipitation indices over the Qilian Mountains in the historical period relatively well, and the corrected CESM2 displayed better simulation as compared to the other two CMIP6 models. The CMIP6 models performed well while simulating R10mm (CC is higher than 0.71) and PRCPTOT (CC is higher than 0.84). (2) The changes in the eight extreme precipitation indices were greater with the enhancement of the SSP scenario. The growth rate of precipitation in the Qilian Mountains during the 21st century under SSP585 is significantly higher than the other two SSP scenarios. The increment of precipitation in the Qilian Mountains mainly comes from the increase in heavy precipitation. (3) The Qilian Mountains will become wetter in the 21st century, especially in the central and eastern regions. The largest increase in precipitation intensity will be observed in the western Qilian Mountains. Additionally, total precipitation will also increase in the middle and end of the 21st century under SSP585. Furthermore, the precipitation increment of the Qilian Mountains will increase with the altitude in the middle and end of the 21st century. This study aims to provide a reference for the changes in extreme precipitation events, glacier mass balance, and water resources in the Qilian Mountains during the 21st century.

## 1. Introduction

In the past decades, global warming has had a profound impact on the climate, not only in terms of the average climate but also in terms of extreme events in different regions of the world [1]. The Intergovernmental Panel on Climate Change (IPCC) sixth assessment report states that global warming will continue and that addressing the challenges caused by climate change due to human influence has become a major issue of the 21st century [2]. Some studies show that the frequency of global extreme precipitation events increases significantly due to global warming [3,4,5]. However, due to different climatic conditions and geographical factors in different regions around the world, these regions are likely to face different challenges due to extreme precipitation events. Therefore, studying regional extreme precipitation events is crucial [6].

The Qilian Mountains are located at the northeast edge of the Qinghai-Tibet Plateau, which is a sensitive area with respect to climate change. Furthermore, the Qilian Mountains are located in the arid area of northwest China, and precipitation plays a vital role in the ecological environment and hydrologic circulation in the region. In recent decades, precipitation in the Qilian Mountains has increased significantly, the results of ERA5-Land show that the average climate tendency rate of precipitation in the Qilian Mountains reached 15.43 mm/10a from 1980 to 2018 [7]. At the same time, a study showed that extreme precipitation events in arid and humid regions of the world have also increased significantly, and the increases in total and extreme precipitation in dry regions are linearly related to global temperature change [8]. For the Qilian Mountains, extreme precipitation events also exhibited a statistically-increasing trend in recent decades, such as the number of days with heavy precipitation and precipitation intensity, etc. [9]. Meanwhile, the greater increasing trend of extreme precipitation events mainly existed in higher altitudes. Moreover, it is worth noting that the change in extreme precipitation events is more likely to cause serious casualties and property losses than the long-term continuous rise in temperature [10]. Therefore, it is particularly important to accurately simulate and project extreme precipitation in the Qilian Mountains under global warming [11,12].

The global climate model is an important tool for studying the mechanism of change in the climate and predicting any future changes in the climate system [13]. It has become an indispensable part of contemporary climate research [1]. The model results of the Coupling Model Comparison Program (CMIP) serve as an important basis for the IPCC assessment report. It is of great significance to the study of ancient climate, the prediction about climate change in the future, the formulation of government decisions, and for signing intergovernmental agreements [14]. Based on the CMIP model, many studies on extreme precipitation events have been carried out in different regions, such as China [15], Australia [16], East Asia [17], the Indochina Peninsula, and South China [18]. These efforts mainly focus on the projection of extreme precipitation indices in the future by using multi-model ensembles and weighted average methods based on model assessments. However, there are still a lot of uncertainties in model projections at the regional and global scales, which include the uncertainty of models. At the same time, a large number of evaluation studies show that any model has its own limitations, especially in high-altitude mountainous areas with complex terrain [7,19]. Therefore, the applicability of these models in the region must be evaluated before using them.

The Scenario Model Intercomparison Project (Scenario-MIP) is the primary activity within Phase 6 of CMIP (CMIP6) that will provide multi-model climate projections based on alternative scenarios of future emissions and land use changes produced with integrated assessment models. In this study, we used three models of CMIP6 to project the future extreme precipitation in the Qilian Mountains. However, the climate projection ability of the model cannot be changed essentially through the method of multimodal integration when models with poor simulation abilities are used. Therefore, we try to use a bias correction method to correct the initial precipitation outputs of CMIP6 models so as to improve the accuracy of future projections of extreme precipitation indices. Besides, the spatial resolution of these models cannot meet the requirements of delicately describing the trend of extreme precipitation in the future. Therefore, we need to downscale the initial outputs of CMIP6 and correct the downscaling results. Quantile delta mapping (QDM) is used for the correction of downscaling results, and this method is widely used in the correction of model results [20,21]. The extreme precipitation indices are calculated by using the meteorological software (ClimPACT2). In this study, the future projections of eight extreme precipitation indices in the Qilian Mountains are made.

The main purpose of this paper is to make future projections of extreme precipitation indices over the Qilian Mountains during the 21st century. At the same time, we analyzed the changes in extreme precipitation indices in the middle (2041–2060) and the end (2081–2100) of the 21st century relative to the base period (1981–2010). In addition, we evaluated the correction effect of the QDM method on the extreme precipitation indices of the models. This study is based on the assumption that better consistency with the current climate indicates that the future prediction of the model is more reliable [22]. On the basis of the above correction effect evaluation, the future projections of extreme precipitation over the Qilian Mountains are shown. Finally, we have also discussed the relationship between future precipitation changes and altitude, as well as the shortcomings of this study. This paper is structured as follows. The data and methods are described in Section 2. In Section 3, an assessment of the spatial interpolation results of station observed data has been presented, and the extreme precipitation indices calculated on the corrected CMIP6 model precipitation have been evaluated. The future projections of the eight indices in the Qilian Mountains under different Shared Socioeconomic Pathway (SSP) scenarios for the 21st century have been presented in Section 4. In Section 5, we give some relevant discussions. The last section is the conclusion.

## 2. Data and Methods

### 2.1. Description of the Study Area

**Figure 1:**
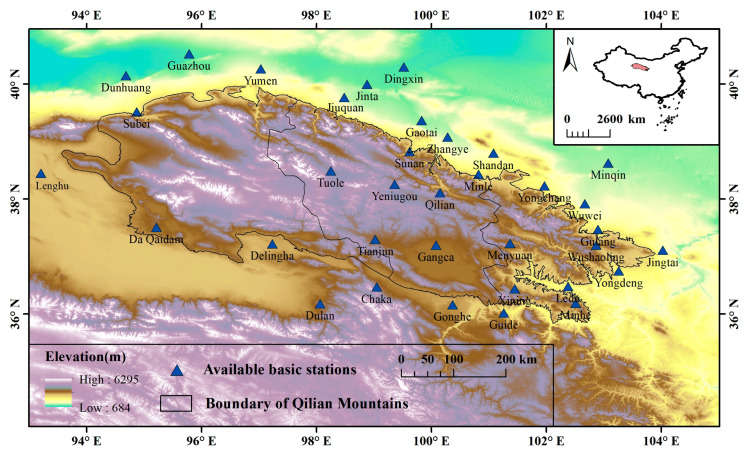
Map of the study area and location of 35 weather stations.

The Qilian Mountains (Figure 1) are located within 36–40° N, 94–104° E. The Qilian Mountains are a part of the northeastern margin of the Qinghai-Tibet Plateau, and it is a sensitive area to climate change. Meanwhile, it is also an important ecological security barrier in northwest China. The data from the observation stations show that the average annual precipitation of the Qilian Mountains from 1970 to 2014 is about 262 mm. A large number of glaciers are distributed in the high-altitude mountains of the Qilian Mountains, with a total ice volume of about 84.48 km^3^, which plays an important role in regulating the runoff of various basins in the region [23]. Many inland rivers originate from the Qilian Mountains [24], and glacier meltwater plays an important role in regulating the runoff of the basin in the region. However, precipitation has an important impact on glacier ablation, surface runoff, ecological environment, etc. In particular, extreme precipitation has a more significant impact on the plateau vegetation and flood disasters in the region. In recent decades, the increase in precipitation in arid areas of northwest China has been due to increased precipitation frequency and intensity [25]. Therefore, it is of great significance to project the future extreme precipitation indices over the Qilian Mountains.

### 2.2. Observations and CMIP6 Model Outputs

The observed data pertaining to daily precipitation records at 35 meteorological stations (Table A1) in the Qilian Mountains and its surroundings was provided by the China Meteorological Administration Meteorological Data Center (http://data.cma.cn/, accessed on 10 February 2020) for the period of 1970–2014. The observation data of stations numbered 1–28 were used as forcing data; they were interpolated to each grid using the gradient and inverse weighted methods in the region [26]. The observation data of stations numbered 29–35 were used to compare and verify the above spatial interpolation results. The grid data of the spatial interpolation results obtained by the above methods were finally used as the observation data (OBS) of the whole Qilian Mountains.

For accuracy, three CMIP6 models (Table 1) were used in this study. The choice of these models was based on an assessment of CMIP6 in simulating precipitation over arid Central Asia [27]. The precipitation dataset of the Climate Research Unit TS 4.04 (CRU, 0.5° × 0.5°) was used as a reference to evaluate the precipitation output from 30 Global Circulation Models (GCMs) of CMIP6 in Hexi Corridor from 1951 to 2014. The results showed that CESM2, EC-Earth3, and KACE-1-0-G performed comparatively well in the region. However, the spatial resolution of these models is relatively rough compared to the complex terrain of the Qilian Mountains. Therefore, we used a spatial interpolation method (Distance-weighted average remapping) to interpolate the model data to a higher spatial resolution (0.1° × 0.1°). At the same time, in order to calculate the extreme precipitation indices of the region more accurately, we adopted an improved quantile mapping bias correction algorithm to correct the systematic distributional biases in CMIP6 model precipitation outputs. We used this method to correct the precipitation output of the model in the historical period and apply this correction relationship to the precipitation output of the model in the future period. We evaluated the ability of the corrected CMIP6 models to simulate the extreme precipitation indices in the Qilian Mountains from 1970 to 2014. The SSP is a combination of the RCP and alternative pathways of socioeconomic growth [28,29]. In addition, the future projection of extreme precipitation indices is conducted under three SSPs (SSP126, SSP245, and SSP585). SSP126, SSP245, and SSP585 represent radiative forgings stabilized at 2.6, 4.5, and 8.5 Wm^−2^ by the late 21st century, respectively. In this study, EC-Earth3 and KACE-1-0-G used the first version (r1i1p1f1), while CESM2 used another version (r11i1p1f1).

### 2.3. Extreme Precipitation Indices

In this study, we selected eight extreme precipitation indices considering the basic climatic characteristics of the Qilian Mountains. At the same time, these extreme precipitation indices are also generally recommended by the Expert Team on Climate Change Detection and Indices (ETCCDMI) [30,31]. These extreme precipitation indices are widely used in relevant research [32,33,34]. These extreme precipitation indices were calculated by using the ClimPACT2 software (https://github.com/ARCCSS-extremes/climpact2/) (accessed on 10 October 2022), and the details of these extreme precipitation indices are displayed in Table 2. In these indices, some were calculated on the basis of station-related thresholds, while others on the basis of fixed thresholds or absolute peak values. When calculating these indices, we uniformly set the base period as 1981–2010. It should be especially noted that some of the indices recommended by ETCCDMI are not relevant for the arid region of China. R99p (extremely wet days) was not analyzed because of multiple zeros in the index for this arid region. For future projections of the extreme precipitation indices, we have used the average changes of the three corrected CMIP6 models.

### 2.4. Bias Correction Algorithm

The quantile mapping (QM) bias correction algorithms are usually used to correct systematic distributional biases in precipitation outputs of climate models. The quantile delta mapping (QDM) used in this study is an improved bias correction algorithm that explicitly preserves relative changes in precipitation quantiles [20]. And the research showed that the QDM algorithm performed well in modifying the trend of mean precipitation and extreme precipitation indices of the global climate model (GCM). The research results of correcting the earth system model of Beijing Normal University (BNU-ESM) by QDM and bias correction constructed analogs with quantile mapping reordering (BCCAQ) show that the QDM has a better performance compared to the BCCAQ method [35]. A study examined the performance of QDM in correcting the deviation of annual maximum daily precipitation, and the results showed that the QDM successfully adjusted the empirical cumulative distribution of climate change projections, removing the systematic error of raw data [36]. And the QDM also presented a suitable performance when applied to future projections. We selected the QDM due to its ability to correct bias in extreme quantiles of wet days. This method is widely used in the correction of climatic elements in the field of geosciences [37,38].

QDM preserves model-projected relative changes in quantiles. At the same time, the systematic deviation in the quantiles of the model output compared with the observed results is corrected. QDM is an improvement based on QM, preservation of relative changes follows directly from the quantile delta change [39] and quantile perturbation [40] methods. The algorithm is divided into two steps: first, future precipitation is de-trended by the quantile, and the simulated values are bias-corrected through quantile mapping while the transfer function in the calibration period is constructed; second, the model-projected relative changes in quantiles are multiplied by the bias-corrected model outputs to obtain the final results. In order to express the algorithm more clearly, we have explained each formula step by step as follows.

QM equates the cumulative distribution function (CDF) between CMIP6 outputs ($F_{m,h}$) and observation data ($F_{o,h}$), the transfer function is as follows,

(1) $${\hat{x}}_{m,p}\left(t\right)=F_{o,h}^{-1}\left\{F_{m,h}\right.\left.\left[x_{m,p}\left(t\right)\right]\right\}$$

where ${\hat{x}}_{m,p}$ is the correction bias for the CMIP6 projection at time $t_{}$, $x_{m,h}$ is historical model data, and $x_{o,h}$ is historical observation data.

If the model projection value is outside the historical period, then some form of extrapolation is required, for example, using parametric distributions following [41] or the constant correction approach of [42], then the equation becomes,

(2) $${\hat{x}}_{m,p}\left(t\right)=F_{o,h}^{-1}\left\{F_{m,h}\left[\frac{{\overset{-}{x}}_{m,h}x_{m,p}\left(t\right)}{{\overset{-}{x}}_{m,p}\left(t\right)}\right]\right\}\frac{{\overset{-}{x}}_{m,p}\left(t\right)}{{\overset{-}{x}}_{m,h}}$$

where ${\overset{-}{x}}_{m,h}$ is the mean CMIP6 output of the historical period, while ${\overset{-}{x}}_{m,p}(t)$ represents the average CMIP6 output at time $t_{}$ in the projected period $p_{}$.

The basic idea of QDM is to maintain relative changes in climate simulation models. QDM starts with the time-dependent CDF of the model series $x_{m,p}$ in the future, for example, as estimated from the empirical CDF around $t_{}$:

(3) $$\tau_{m,p}\left(t\right)=F_{m,p}^{\left(t\right)}\left[x_{m,p}(t)\right],\tau_{m,p}(t)\in \left\{0,1\right\}$$

where $\tau_{m,p}(t)$ is the non-exceedance probability associated with the value at time $t_{}$. The historical modeled $\tau_{m,p}(t)$ quantile can be found by entering this value into the inverse CDF in the historical period. The relative quantile change between the historical period and future time $t_{}$ is calculated by

(4) $${∆}_{m}\left(t\right)=\frac{F_{m,p}^{\left(t\right)-1}\left[\tau_{m,p}(t)\right]}{F_{m,h}^{-1}\left[\tau_{m,p}(t)\right]}=\frac{x_{m,p}(t)}{F_{m,h}^{-1}\left[\tau_{m,p}(t)\right]}$$

The CMIP6 model $\tau_{m,p}$ quantile at time $t_{}$ can be corrected by applying the inverse CDF estimated from the historical observation $x_{o,h}$,

(5) $${\hat{x}}_{o:m,h:p}\left(t\right)=F_{o,h}^{-1}\left[\tau_{m,p}(t)\right]$$

The corrected model outputs in the future at time $t_{}$ is given by applying the model-projected relative change ${∆}_{m}(t)$ multiplied by this bias corrected CMIP6 outputs,

(6) $${\hat{x}}_{m,p}\left(t\right)={\hat{x}}_{o:m,h:p}(t){∆}_{m}(t)$$

### 2.5. Model Evaluation Metrics

#### 2.5.1. Statistical Metrics

To evaluate the accuracy of spatial interpolation, the Kling–Gupta efficiency (KGE) was used to quantify the daily differences between the observations and interpolation results in this study. At the same time, in order to evaluate the accuracy of extreme precipitation indices calculated by the corrected CMIP6 model, we have used some error evaluation indices (the correlation coefficient (CC), root mean square error (RMSE), mean absolute error (MAE), and relative bias (BIAS)). These statistical metrics have been widely used in similar evaluation studies [43,44]. Their specific formulas are as follows:

(7) $$KGE=1-\sqrt{{\left(CC-1\right)}^{2}+{\left(\beta -1\right)}^{2}+{\left(\gamma -1\right)}^{2}}\;KGE\in (-\infty ,1]$$

$$\beta =\frac{\overset{-}{S}}{\overset{-}{O}},\;\gamma ={CV}_{s}/{CV}_{o}$$

(8) $$CC=\frac{\sum_{i=1}^{n}\left(S_{i}-\overset{-}{S}\right)\left(O_{i}-\overset{-}{O}\right)}{\sqrt{\sum_{i=1}^{n}{\left(S_{i}-\overset{-}{S}\right)}^{2}}\sqrt{\sum_{i=1}^{n}{\left(O_{i}-\overset{-}{O}\right)}^{2}}}\;CC\in [-1,1]$$

(9) $$RMSE=\sqrt{\frac{1}{n}\sum_{i=1}^{n}{\left(S_{i}-O_{i}\right)}^{2}}\;RMSE\in [0,+\infty )$$

(10) $$MAE=\frac{1}{n}\sum_{i=1}^{n}\left|S_{i}-O_{i}\right|\;MAE\in [0,+\infty )$$

(11) $$BIAS=\frac{\sum_{i=1}^{n}\left(S_{i}-O_{i}\right)}{\sum_{i=1}^{n}O_{i}}\times 100\;BIAS\in (-\infty ,+\infty )$$

In the formulas, $S_{i}$ is the value of spatial interpolation at time $i_{}$, and $O_{i}$ is the value observed by the weather station. $\overset{-}{S}$ and $\overset{-}{O}$ are the average values during the corresponding periods, and *CV* is the coefficient of variation. We calculated these error metrics for stations numbered 29–35 during the period of 1970–2014.

#### 2.5.2. Taylor Diagram

The Taylor diagram is widely used to compare the model outputs with observations, and the commonly used accuracy indicators are correlation coefficient, standard deviation, and root mean square deviation (RMSD) [45]. In this study, the ability of the CMIP6 model to simulate the extreme precipitation in the Qilian Mountains from 1970 to 2014 was quantitatively evaluated using the Taylor diagram. Scattered points in the Taylor diagram represent the CMIP6 models, the radiation line represents the correlation coefficient between the models and the observations, the horizontal and the vertical axes represent the standard deviation, and the dotted line represents the root mean square deviation.

#### 2.5.3. Inter-Annual Variability Skill Score

In order to evaluate the ability of the corrected CMIP6 model to simulate the inter-annual variation of extreme precipitation indices in the Qilian Mountains, we used the inter-annual variability skill (IVS) score [46]. The skill score IVS is expressed as:

(12) $$IVS={\left(\frac{{STD}_{m}}{{STD}_{o}}-\frac{{STD}_{o}}{{STD}_{m}}\right)}^{2}$$

where ${STD}_{m}$ and ${STD}_{o}$ are the inter-annual standard deviations of the corrected CMIP6 model outputs and observed values, respectively. The IVS score of each index was first calculated at the grid point corresponding to each station, and then the average value of 35 stations was calculated. IVS is a symmetric variability statistic, and the smaller IVS score indicates that the inter-annual variability between the CMIP6 model simulations and observed values is more consistent.

## 3. Model Evaluation

In this section, we use an index (KGE) to evaluate the accuracy of the spatial interpolation results of the station observation data. Furthermore, we mainly evaluated the simulation performance of the corrected CMIP6 model on the extreme precipitation indices of the Qilian Mountains from 1970 to 2014.

### 3.1. Evaluation of the Spatial Interpolation

**Figure 2:**
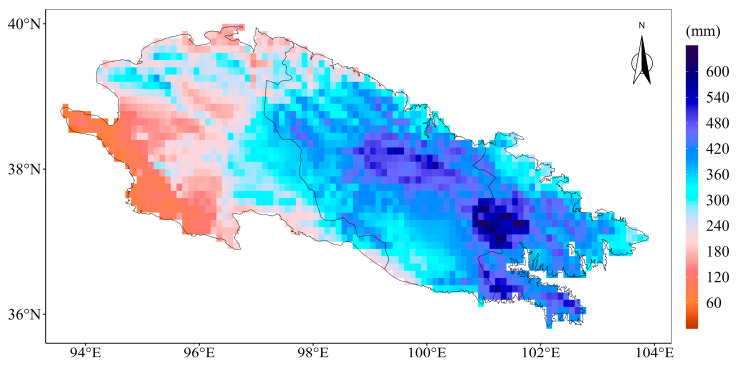
The spatial distribution of the annual average precipitation in the Qilian Mountains from 1970 to 2014 based on the spatial interpolation results.

**Figure 3:**
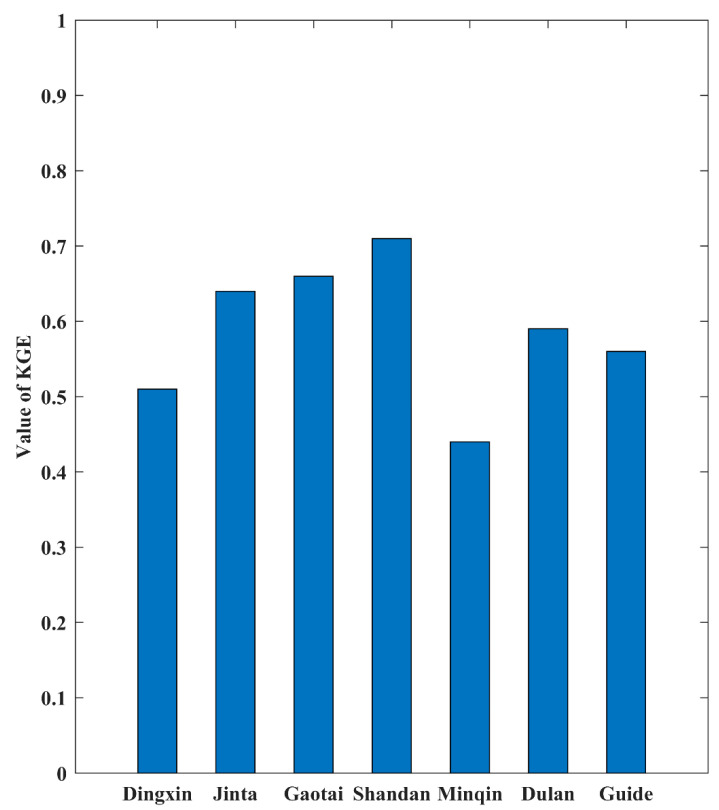
The Kling–Gupta efficiency (KGE) of spatial interpolation at seven weather stations.

Due to the complex terrain conditions of the Qilian Mountains, it is necessary to first evaluate the spatial interpolation results. In order to intuitively express the ability of the interpolation results to depict the spatial pattern of precipitation in the Qilian Mountains, the spatial distribution of the annual average precipitation in the Qilian Mountains from 1970 to 2014 based on the spatial interpolation results is shown in Figure 2. It can be seen from Figure 2 that the precipitation in the Qilian Mountains generally presents a spatial distribution pattern of decreasing from east to west and from the central mountains to the surrounding low-altitude areas, which is consistent with previous research results [7]. KGE is a comprehensive index of error evaluation, which is used to reflect the simulation performance of interpolation results with respect to the daily precipitation. Figure 3 shows the simulation error of interpolation results for daily precipitation at seven observation stations. The average value of KGE is 0.587 (the maximum value is 0.71, and the minimum value is 0.44), and these results show that the spatial interpolation method adopted in this paper has relatively good simulation performance for daily precipitation in the Qilian Mountains. Additionally, the spatial interpolation result is an important basis for the correction of model precipitation in the Qilian Mountains.

### 3.2. Evaluation of Spatial Variation in Extreme Precipitation Indices

**Figure 4:**
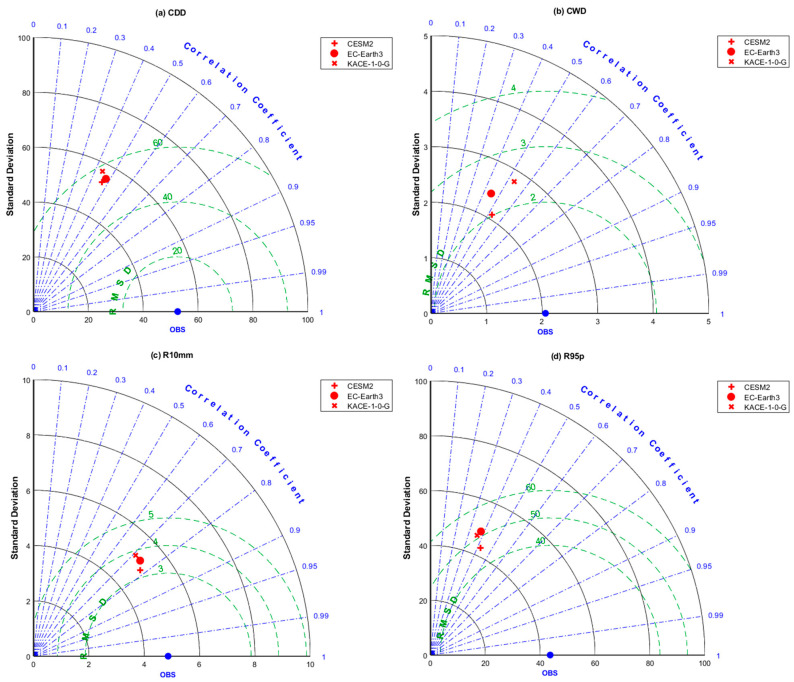
Taylor diagrams indicating correlation coefficients, standard deviation, and RMSE of CDD, CWD, R10mm, and R95p from 1970 to 2014.

**Figure 5:**
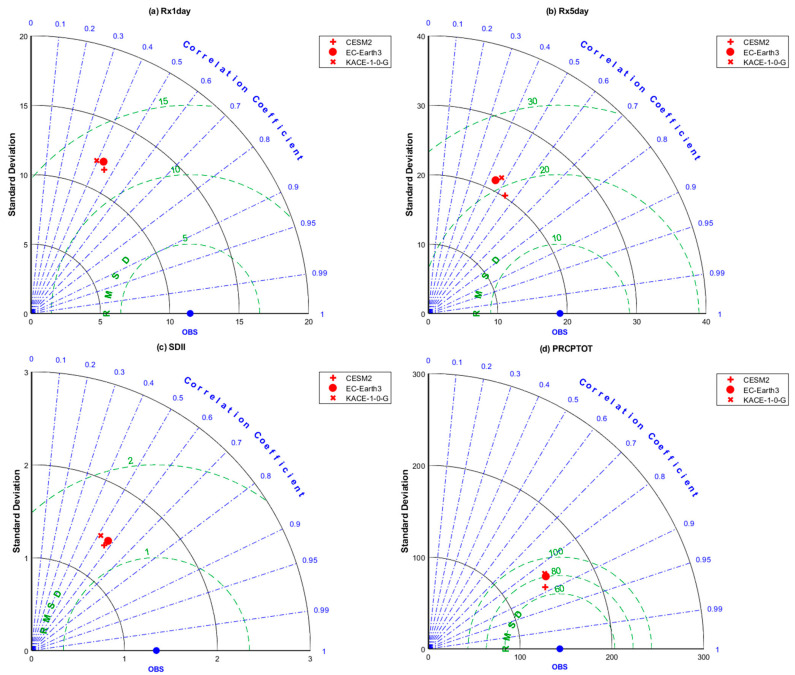
Taylor diagrams indicating correlation coefficients, standard deviation, and RMSE of Rx1day, Rx5day, SDII, and PRCPTOT from 1970 to 2014.

In order to comprehensively reflect the simulation performance of the CMIP6 models on the spatial variation of extreme precipitation indices, we use the Taylor diagram to visually present the evaluation results. We evaluated the ability of each model to simulate the eight extreme precipitation indices at 35 observation stations in the Qilian Mountains. Figure 4 and Figure 5 display the ability of the corrected CMIP6 model (hereinafter referred to as the CMIP6 model) results to simulate the spatial changes of the eight extreme precipitation indices in the Qilian Mountains. Figure 4 reveals that CESM2 could well simulate the four extreme precipitation indices (CDD, CWD, R10mm, and R95p) in the Qilian Mountains. However, there is no significant difference in the simulation performance among the three CMIP6 models. And they have the best simulation performance for R10mm. Figure 5 shows the simulation ability of the spatial changes with regard to the remaining four extreme precipitation indices (Rx1day, Rx5day, SDII, and PRCPTOT). CESM2 exhibited relatively good performance as compared to the other two CMIP6 models. The best extreme precipitation index simulated by the three CMIP6 models in the Qilian Mountains is PRCPTOT, and the correlation coefficient between the model results and the observed values exceeds 0.8. Table 3 displays the statistical errors of the eight extreme precipitation indices between the original CMIP6 models and the observed values. It can be seen from Table 3 that the extreme precipitation indices calculated based on the original precipitation outputs of the CMIP6 models have a large deviation. Table 4 shows the detailed statistical errors of eight indices between the corrected CMIP6 models and the observations. Table 4 indicates that CESM2 performs better than the other two models in simulating extreme precipitation indices. However, no significant differences in the simulation of extreme precipitation in this area were observed among the three models. The comparison results in Table 3 and Table 4 show that the correction of the CMIP6 model by QDM has significantly improved the simulation performance of the model for extreme precipitation indices, especially in BIAS. The three corrected CMIP6 models perform relatively well in simulating the spatial pattern of extreme precipitation over the Qilian Mountains, having complex terrain.

### 3.3. Evaluation of Inter-Annual Variability in Extreme Precipitation Indices

**Figure 6:**
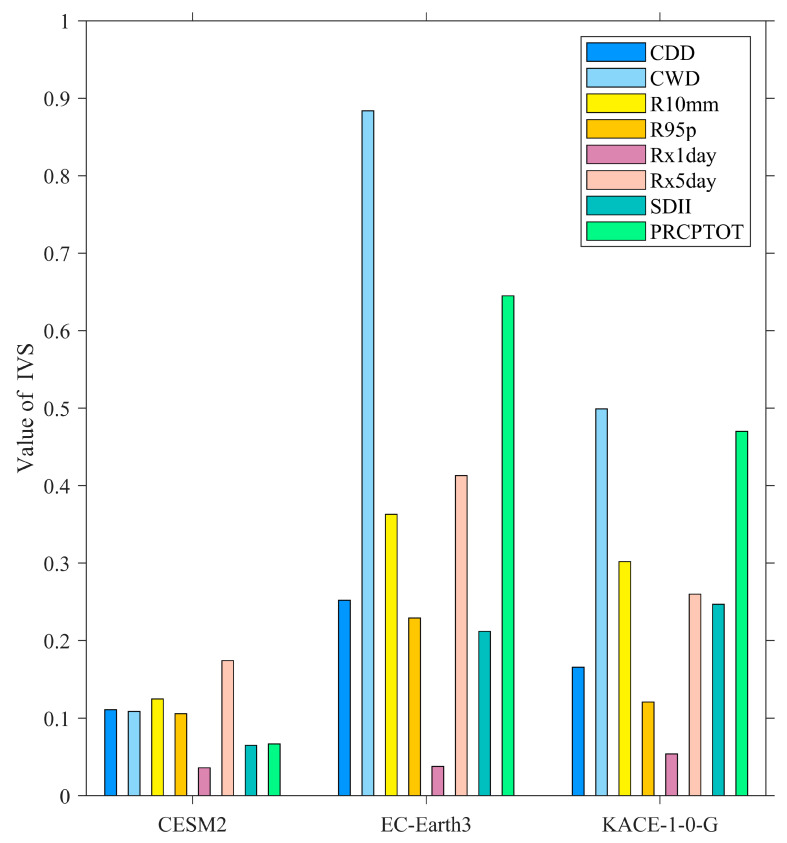
The IVS scores of the corrected CMIP6 models for eight extreme precipitation indices over the Qilian Mountains.

The ultimate purpose of this study is to project the future extreme precipitation indices of the Qilian Mountains. Therefore, it is particularly important for the CMIP6 model to possess the ability to simulate the inter-annual changes in the extreme precipitation indices. The CC between the PRCPTOT simulated by CESM2 and the observed results is 0.88 (Table 4), indicating that the model can well simulate the inter-annual variation of PRCPTOT. The CMIP6 models also have a good simulation for the tendency of R10mm. The relatively poor extreme precipitation indices simulated by the models are CDD, R95p, and Rx1day, and their CC values are less than 0.5. Figure 6 shows the IVS scores of the eight indices over the Qilian Mountains from 1970 to 2014. In general, CESM2 has the best ability to simulate the inter-annual variations of extreme precipitation indices in the Qilian Mountains, followed by KACE-1-0-G and EC-Earth3, which exhibited the worst performance. The IVS scores of all the extreme precipitation indices of CESM2 are below 0.2, which indicates that this model exhibited comprehensive excellent performance in simulating the inter-annual variation of the extreme precipitation indices in the Qilian Mountains. However, all IVS scores of EC-Earth3 and KACE-1-0-G are below 0.9, and they also perform relatively well compared with other relevant studies. Rx1day performed best in all the extreme precipitation indices, while CWD exhibited the worst performance. This may be attributed to the lower ability of the model to catch small precipitation in high-altitude mountainous areas. In addition, the performance of Rx5day and PRCPTOT simulated by EC-Earth3 and KACE-1-0-G in inter-annual change is also relatively poor. This result also shows that the model has a limited ability to depict small and large precipitation in high-altitude mountainous areas. Overall, the three corrected CMIP6 model outputs could well simulate the inter-annual variations of the extreme precipitation indices in the Qilian Mountains.

## 4. Future Projection

In this section, we explore the future changes in the eight extreme precipitation indices over the Qilian Mountains. In order to more concisely express the future changes in extreme precipitation, we mainly discuss the changes in indices in the middle and end of the 21st century. At the same time, our projection is conducted under the three SSPs to compare the future changes in extreme precipitation indices under different SSP scenarios.

### 4.1. Future Projection of the Eight Extreme Precipitation Indices

**Figure 7:**
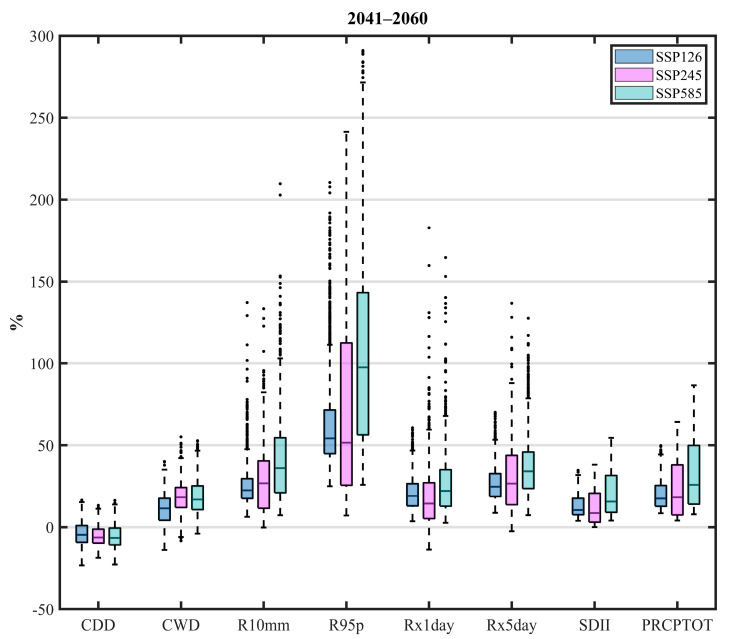
Box-and-whisker plots for the projected changes (expressed as percentages) of eight extreme precipitation indices in the period 2041–2060 as compared to the base period under three SSPs over the Qilian Mountains. The upper and lower edges of each box represent the 75th and 25th percentile values, respectively, while the line in each box shows the median of the distribution. The upper and lower whiskers represent the upper and lower limits of the normal range, respectively. Note that the future change is represented by the ensemble of three corrected CMIP6 models.

**Figure 8:**
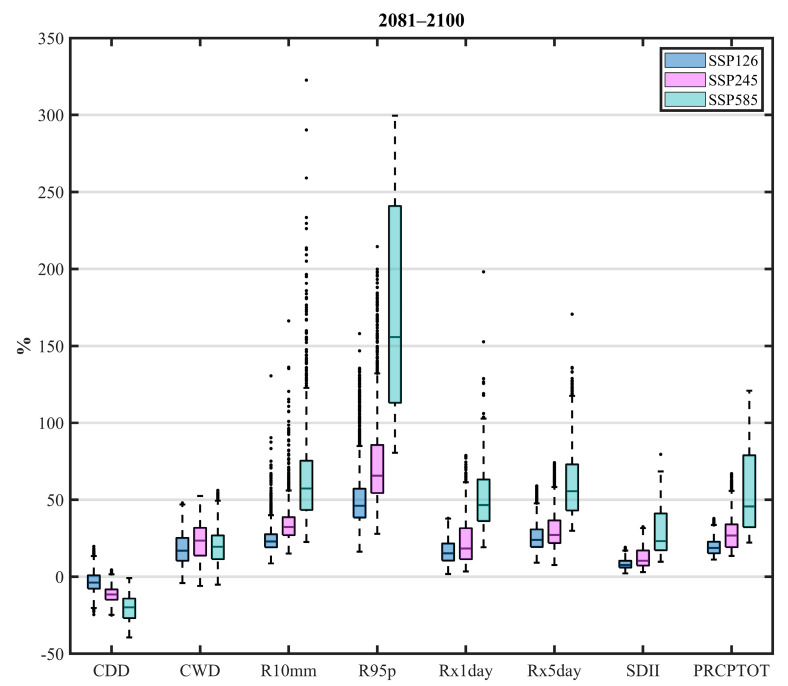
As in Figure 7, but for the period 2081–2100.

The future projections of the eight indices in the middle and late of the 21st century compared with the historical period (1981–2010) over the Qilian Mountains are shown in Figure 7 and Figure 8. In order to depict the future changes in extreme precipitation indices uniformly and intuitively, the projected changes are expressed as percentages. First, we calculated the average values of the eight extreme precipitation indices at each grid point in different periods and then calculated the relative change of the future compared with the historical period under different SSPs. The future changes of the eight indices are presented in the form of a box-and-whisker plot. In addition, the black dots beyond the upper and lower whiskers in the boxplot represent abnormal values, which may be caused by the small values in the historical period. Note that the results shown in Figure 7 and Figure 8 are from the three CMIP6 models with the same weight, and the future change is represented by the ensemble of these models. And this is to reduce the possibility that a single model will cause a large deviation in one aspect. Figure 7 indicates that the drought index (CDD) in most areas of the Qilian Mountains is decreasing in the middle of the 21st century. Meanwhile, the wet index (CWD) will increase significantly due to climate warming, and the range of change exceeds the drought index. The increase in the remaining seven indices is almost certain during the mid-21st century (Figure 7). R95p shows the largest growth, while the change of CDD is relatively slow as compared to the other seven indices. At the same time, in combination with PRCPTOT, the increase in precipitation over the Qilian Mountains mainly comes from the increase in heavy precipitation. Besides, the changes in other indices (R10mm, Rx1day, and Rx5day) representing precipitation intensity also verified that the intensity and the days of heavy precipitation in the Qilian Mountains increased by the middle of the 21st century. Furthermore, the SDII shows that the average precipitation intensity on rainy days is also likely to increase significantly. However, the medians of some indices (R95p, Rx1day, and SDII) under SSP245 are lower than that under SSP126. This shows that the overall precipitation intensity of the Qilian Mountains under SSP245 is weaker than that under SSP126, but the number of precipitation days is likely to increase.

The changes in the eight extreme precipitation indices at the end of the 21st century as compared to the historical period are shown in Figure 8. The changes in extreme precipitation indices in the late 21st century are similar to that in the middle of the 21st century, but the amplitude of change is larger. At the same time, there are greater differences between the scenarios at the end of the 21st century. For the end of the 21st century, the drought index (CDD) of the Qilian Mountains is likely to be significantly lower than that in the middle of the 21st century, but the relative changes of the wet index (CWD) in the two periods are very similar. In general, the Qilian Mountains are likely to become wetter during the 21st century. Some extreme precipitation indices (R10mm, R95p, Rx1day, Rx5day, and SDII) indicating precipitation intensity show that the intensity and days of heavy precipitation in the Qilian Mountains will increase significantly under SSP585. Hence, the total precipitation of the Qilian Mountains will also increase significantly, which can be inferred from the change in PRCPTOT. R95p is the index with the largest change in the middle and the end of the 21st century. In particular, the median change of R95p under SSP585 at the end of the 21st century is likely to exceed 150%. This change indicates that the threshold of extreme precipitation in the Qilian Mountains will increase significantly under the background of a warmer climate. This will greatly increase the risk of flood disasters in the Qilian Mountains and may also increase the possibility of geological disasters in the region. It can be seen from Figure 8 that the extremely heavy rainfall at the end of the 21st century will yield unprecedented challenges for the Qilian Mountains.

Table 5 shows the average changes in the eight extreme precipitation indexes in the Qilian Mountains under different SSPs. Under the SSP126 scenario, CDD at the end of the 21st century will increase by 0.68 days on average as compared to that in the middle of the 21st century. CWD under different SSPs will continue to increase in the 21st century. This indicates that the Qilian Mountains will become wetter in the 21st century due to the higher Shared Socioeconomic Pathway scenario. The R10mm shows that the number of days of heavy precipitation in the Qilian Mountains will continue to increase during the 21st century, which is consistent with the changing trend of PRCPTOT. The growth rate of precipitation in the Qilian Mountains during the 21st century under SSP585 is significantly higher than the other two SSP scenarios, while the PRCPTOT of Qilian Mountains will increase by 66.4 mm at the end of the 21st century as compared to the middle of the 21st century. Similarly, the indices (R95p, Rx1day, Rx5day, and SDII) representing precipitation intensity under the SSP585 scenario will also increase significantly at the end of the 21st century.

### 4.2. Spatial Variations of the Future Extreme Precipitation Indices

**Figure 9:**
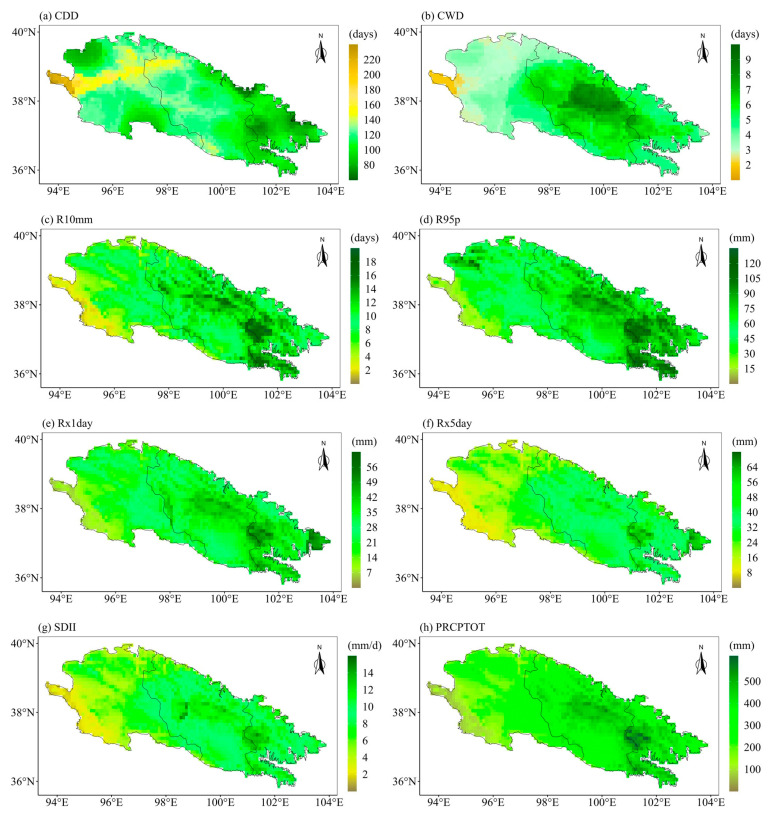
The spatial patterns of eight extreme precipitation indices in the Qilian Mountains during the base period (1981–2010).

**Figure 10:**
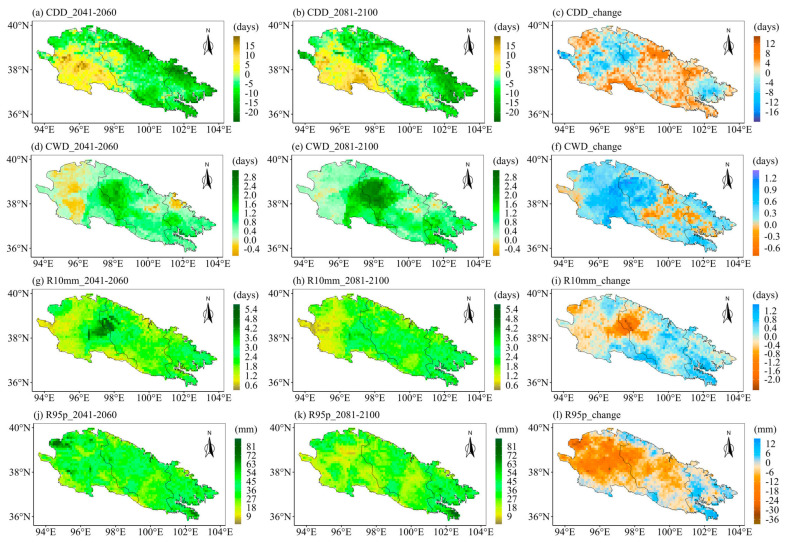
(**left**) The changes of extreme precipitation indices in the middle of the 21st century as compared to the base period, (**middle**) the changes of extreme precipitation indices at the end of the 21st century as compared to the base period, and (**right**) the changes of extreme precipitation indices at the end of the 21st century as compared to the middle of the 21st century. Spatial distribution over the Qilian Mountains under SSP126: (**a**–**c**) CDD, (**d**–**f**) CWD, (**g**–**i**) R10mm, and (**j**–**l**) R95p. Note that the future change is represented by the ensemble of the corrected CMIP6 models.

**Figure 11:**
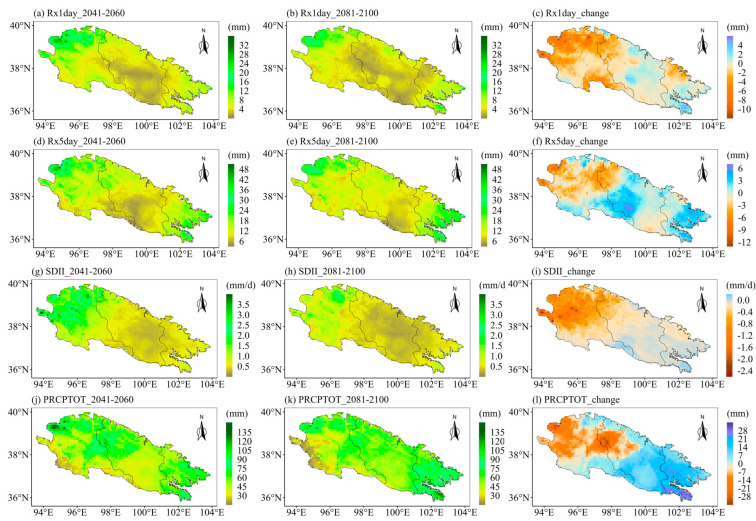
(**left**) The changes of extreme precipitation indices in the middle of the 21st century as compared to the base period, (**middle**) the changes of extreme precipitation indices at the end of the 21st century as compared to the base period, and (**right**) the changes of extreme precipitation indices at the end of the 21st century as compared to the middle of the 21st century. Spatial distribution over the Qilian Mountains under SSP126: (**a**–**c**) Rx1day, (**d**–**f**) Rx5day, (**g**–**i**) SDII, and (**j**–**l**) PRCPTOT. Note that the future change is represented by the ensemble of the corrected CMIP6 models.

**Figure A1:**
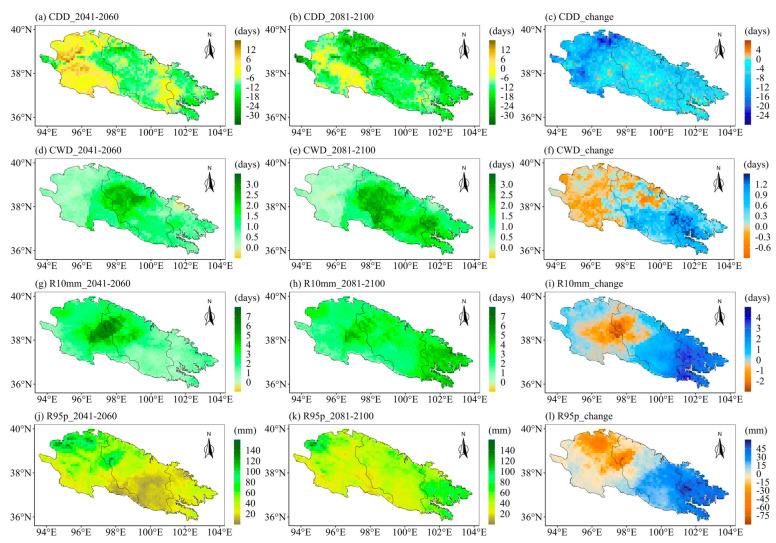
(**left**) The changes of extreme precipitation indices in the middle of the 21st century as compared to the base period, (**middle**) the changes of extreme precipitation indices at the end of the 21st century as compared to the base period, and (**right**) the changes of extreme precipitation indices at the end of the 21st century as compared to the middle of the 21st century. Spatial distribution over the Qilian Mountains under SSP245: (**a**–**c**) CDD, (**d**–**f**) CWD, (**g**–**i**) R10mm, and (**j**–**l**) R95p. Note that the future change is represented by the ensemble of the corrected CMIP6 models..

**Figure A2:**
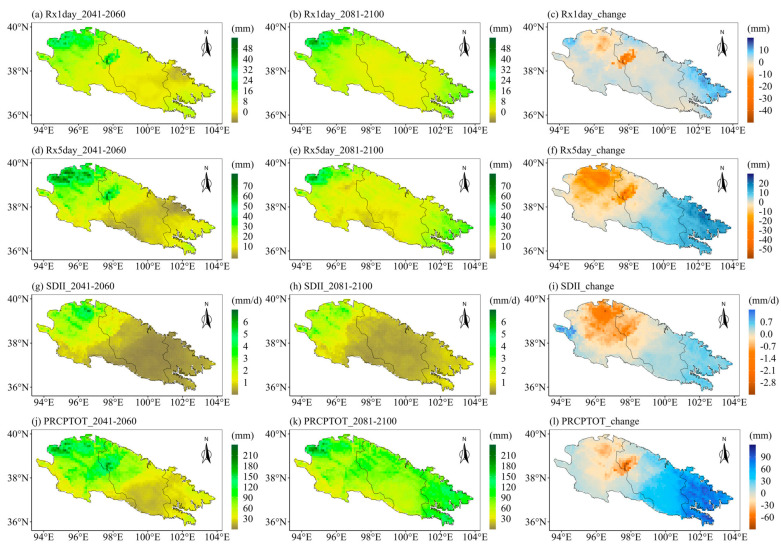
(**left**) The changes of extreme precipitation indices in the middle of the 21st century as compared to the base period, (**middle**) the changes of extreme precipitation indices at the end of the 21st century as compared to the base period, and (**right**) the changes of extreme precipitation indices at the end of the 21st century as compared to the middle of the 21st century. Spatial distribution over the Qilian Mountains under SSP245: (**a**–**c**) Rx1day, (**d**–**f**) Rx5day, (**g**–**i**) SDII, and (**j**–**l**) PRCPTOT. Note that the future change is represented by the ensemble of the corrected CMIP6 models.

The ecological landscape of the Qilian Mountains varies greatly in different regions and altitudes due to its special geographical environment, which means that the sensitivities of different regions of the Qilian Mountains to extreme precipitation are very different. Therefore, it is particularly important to project the future spatial changes in extreme precipitation indices over the Qilian Mountains. Further, we explore the spatial variations of the eight extreme precipitation indices under different SSPs. We first calculated the averages of extreme precipitation indices for different periods at each grid point. Then we calculated the changes in the eight extreme precipitation indices in the future two periods as compared to the historical period. Finally, we displayed the changes in the extreme precipitation indices at each grid point in space. Figure 9 displays the spatial patterns of eight extreme precipitation indices in the Qilian Mountains during the base period (1981–2010). Figure 9a shows the spatial pattern of the Qilian Mountains gradually drying from east to west. The CWD is the largest in the middle of the Qilian Mountains in the base period (Figure 9b). The indices representing precipitation (PRCPTOT) and precipitation intensity (R10mm, R95p, Rx1day, Rx5day, and SDII) show a gradually decreasing spatial pattern from east to west (Figure 9c–h). The spatial change patterns of the eight indices of the Qilian Mountains under different SSPs are similar on the whole, but the magnitudes are different, especially between SSP126 and SSP585. Moreover, the extreme precipitation indices are also likely to vary during different periods in the future. The following only show the results under two scenarios (SSP126 and SSP585), while those under SSP245 are shown in Appendix A (see Figure A1 and Figure A2). The first column of each figure shows the changes in each extreme precipitation index in the middle of the 21st century relative to the historical period. The second column of each figure shows the changes in the extreme precipitation indices at the end of the 21st century relative to the historical period. The third column of each figure shows the changes in extreme precipitation indices at the end of the 21st century relative to the middle 21st century. Figure 10 and Figure 11 show the spatial variations of the eight indices over the Qilian Mountains under SSP126 in the middle and end of the 21st century. The spatial variation patterns of extreme precipitation indices in Figure 10 and Figure 11 were calculated based on simple multi-model averages. The drought index (CDD) in the southern part of the western Qilian Mountains will increase in the middle and late 21st century under SSP126. And the drought index (CDD) in most areas of the Qilian Mountains will increase at the end of the 21st century as compared to the middle of the 21st century. This pattern is also reflected in the change in the wet index (CWD). However, the wet index (CWD) of the western Qilian Mountains will increase significantly at the end of the 21st century as compared to the middle of the 21st century (Figure 10f). The days of heavy precipitation (R10mm) in the western Qilian Mountains will be significantly reduced at the end of the 21st century compared with the middle of the 21st century (Figure 10i). The 95th percentile threshold of precipitation intensity (R95p) also has a similar change pattern as in R10mm, and its area is larger (Figure 10l). The indices (Rx1day and Rx5day) representing the intensity of single-day precipitation and continuous precipitation will increase in most parts of the Qilian Mountains in the middle and end of the 21st century (Figure 11a,b,d,e). However, Rx1day at the end of the 21st century is significantly lower than that at the middle of the 21st century in most areas of the Qilian Mountains. In addition, the average precipitation intensity (SDII) and total precipitation (PRCPTOT) of precipitation days have similar performance with Rx1day and Rx5day in the middle and end of the 21st century. In general, the number of days of precipitation in the western Qilian Mountains will increase at the end of the 21st century as compared to the middle of the 21st century, but the precipitation intensity and total precipitation are likely to decrease. At the same time, on combining the results of Figure 10 and Figure 11, it can be seen that the increase in precipitation of the Qilian Mountains during the 21st century under SSP126 can mainly be attributed to the increase in precipitation intensity and the number of heavy precipitation days in most areas of the central and eastern Qilian Mountains.

**Figure 12:**
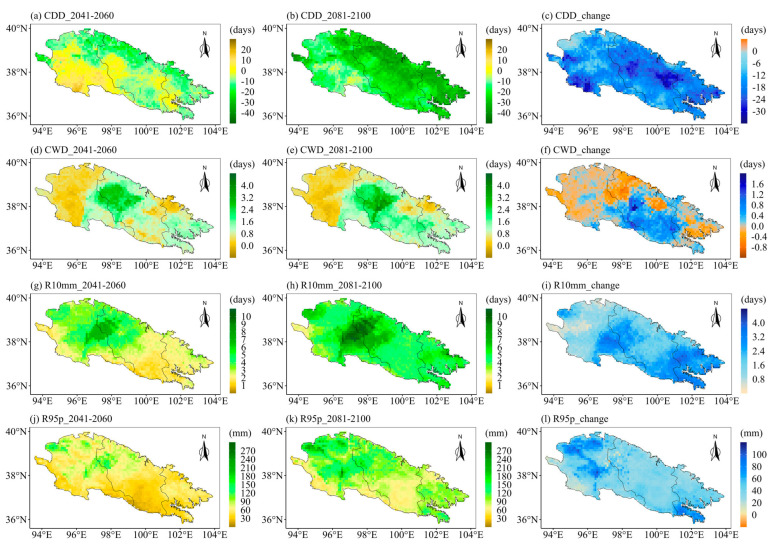
(**left**) The changes of extreme precipitation indices in the middle of the 21st century as compared to the base period, (**middle**) the changes of extreme precipitation indices at the end of the 21st century as compared to the base period, and (**right**) the changes of extreme precipitation indices at the end of the 21st century as compared to the middle of the 21st century. Spatial distribution over the Qilian Mountains under SSP585: (**a**–**c**) CDD, (**d**–**f**) CWD, (**g**–**i**) R10mm, and (**j**–**l**) R95p. Note that the future change is represented by the ensemble of the corrected CMIP6 models.

**Figure 13:**
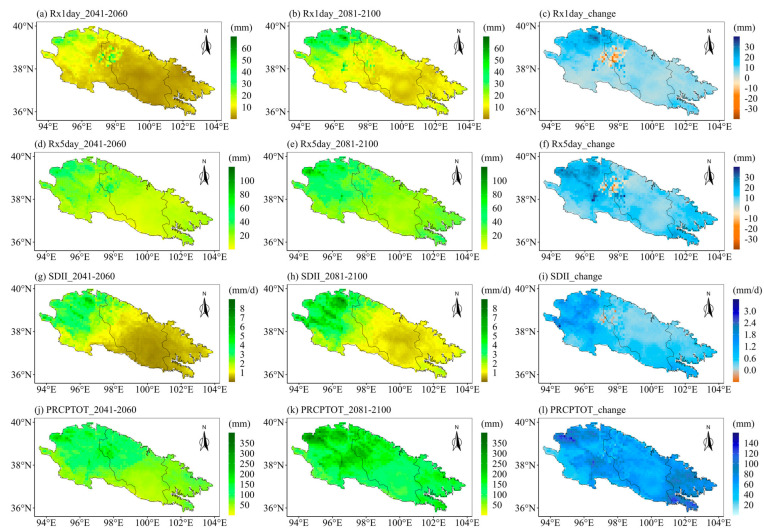
(**left**) The changes of extreme precipitation indices in the middle of the 21st century as compared to the base period, (**middle**) the changes of extreme precipitation indices at the end of the 21st century as compared to the base period, and (**right**) the changes of extreme precipitation indices at the end of the 21st century as compared to the middle of the 21st century. Spatial distribution over the Qilian Mountains under SSP585: (**a**–**c**) Rx1day, (**d**–**f**) Rx5day, (**g**–**i**) SDII, and (**j**–**l**) PRCPTOT. Note that the future change is represented by the ensemble of the corrected CMIP6 models.

Next, we discuss the future spatial changes in eight indices over the Qilian Mountains under the extreme emission scenario SSP585 (Figure 12 and Figure 13). By the end of the 21st century, the drought index (CDD) will have significantly decreased in most parts of the Qilian Mountains. In addition, the CDD in most parts of the Qilian Mountains at the end of the 21st century will also exhibit a significant increase trend as compared to the middle of the 21st century. The most obvious increase of the wet index (CWD) under SSP585 in the middle and end of the 21st century is located in the middle of the Qilian Mountains (Figure 12d,e). The number of heavy precipitation days (R10mm) will also increase significantly at the end of the 21st century, and this growth trend can be observed in any part of the Qilian Mountains (Figure 12g–i). The 95th percentile threshold of precipitation intensity under SSP585 has a very similar spatial variation trend with R10mm. The intensity of precipitation in a single day and continuous precipitation is likely to increase significantly in the middle and end of the 21st century (Figure 13a,b,d,e). In addition, the SDII and PRCPTOT (Figure 13g–l) under SSP585 will exhibit similar trends of spatial changes with Rx1day and Rx5day. It is worth noting that the largest increase in the precipitation intensity and total precipitation will be observed in the western Qilian Mountains in the middle and late of the 21st century under the SSP585 scenario. Additionally, the precipitation intensity and total precipitation of the Qilian Mountains will maintain continuous growth in the middle and end of the 21st century under SSP585, which is different from SSP126.

### 4.3. Inter-Annual Variations of the Future Extreme Precipitation Indices

**Figure 14:**
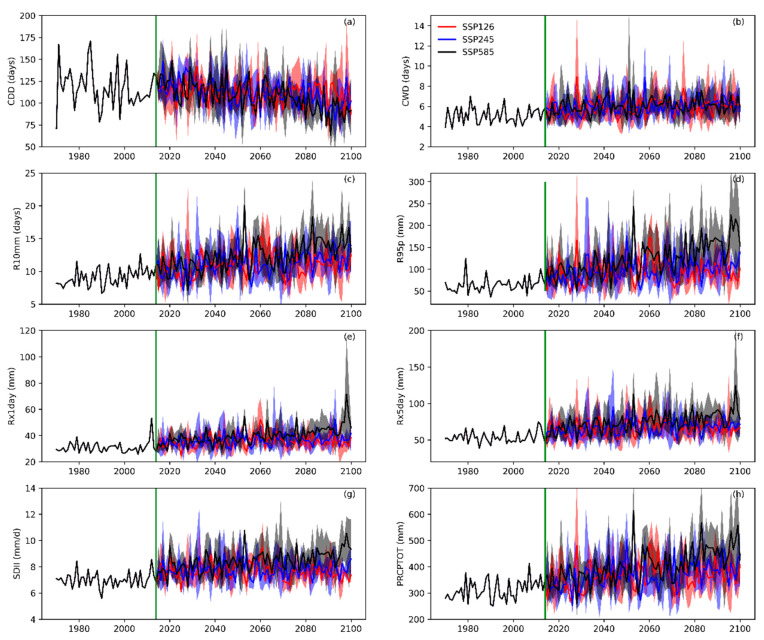
The inter-annual changes of eight extreme precipitation indices in the Qilian Mountains from 1970 to 2100. In the figure, (**a**–**h**) is respectively CDD, CWD, R10mm, R95p, Rx1day, Rx5day, SDII, and PRCPTOT. The green line in the figure (representing 2014) is the dividing line between the historical period and the future.

The Qilian Mountains are the source of many inland river basins in western China, such as the Heihe basin, Shiyanghe basin, Shulehe basin, etc. The inter-annual fluctuation of the extreme precipitation indices over the Qilian Mountains in the 21st century has a direct impact on the downstream runoff change and annual runoff. Therefore, it is very important to project the future inter-annual changes in the extreme precipitation indices over the Qilian Mountains. In order to have an intuitive understanding of the inter-annual changes of the extreme precipitation indices in the Qilian Mountains under different SSPs, the historical and future trends of the eight extreme precipitation indices have been illustrated in Figure 14. We have first calculated the averages of the extreme precipitation indices at all grid points in the Qilian Mountains and have presented their inter-annual changes. It can be seen from Figure 14a that the inter-annual variation in the drought index is strongly volatile both in the historical period and in the future. Additionally, the CDD is likely to follow a fluctuating decrease trend in the 21st century. The remaining seven extreme precipitation indices show a fluctuating upward trend under different SSPs in the 21st century. As compared to the other indices, the CWD shows the most stable and slow change trend (Figure 14b). Figure 14c shows the inter-annual variation trends of R10mm under different SSPs, and the results reveal that the rate of increase in the number of days of heavy precipitation under the SSP585 scenario is significantly higher than the other two scenarios. The 95th percentile threshold of precipitation intensity under SSP585 is also significantly higher than that in the other two scenarios at the end of the 21st century (Figure 14d). At the same time, R95p and other indices (Rx1day, Rx5day, and SDII) representing precipitation intensity have a good consistency with PRCPTOT in the changing trend (Figure 14d–h). Moreover, these indices representing precipitation intensity and total precipitation show significant growth under SSP585, especially during the end of the 21st century.

## 5. Discussion

### 5.1. Comparison with Other Similar Studies

Under the background of global warming, the extreme precipitation indices of the Qilian Mountains will change significantly in the middle and late 21st century. At the same time, a study on the future extreme precipitation in China shows that the extreme precipitation in the Qinghai-Tibet Plateau and the arid and semi-arid areas in western China are highly sensitive to climate warming [47]. In the 21st century, PRCPTOT will increase significantly in the Qilian Mountains, which is consistent with the research results of the Tienshan Mountains in northwest China [48]. And with the enhancement of the SSP scenario, all extreme precipitation indices will change dramatically. The study on the future extreme precipitation in Central Asia shows that the extreme precipitation indices will increase approximately linearly with the enhancement of global warming, except for the continuous dry day (CDD) index [49]. The future change in extreme precipitation indices over the Qilian Mountains also shows a similar pattern to that in Central Asia. On the whole, the future trend of extreme precipitation over the Qilian Mountains in this study is in good agreement with that in the surrounding climate-similar areas.

### 5.2. Relationship between Future Precipitation and Altitude

**Figure 15:**
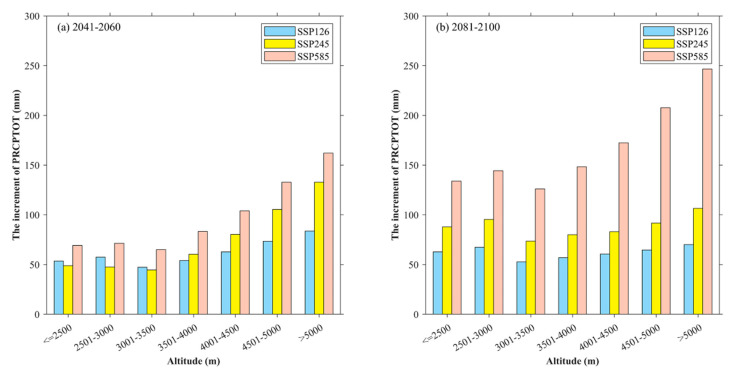
PRCPTOT increments at different altitudes of the Qilian Mountains in the middle and late 21st century compared with the historical period.

The spatial and inter-annual changes in the extreme precipitation indices reveal that the change of PRCPTOT has a direct impact on the trends of other extreme precipitation indices, especially R10mm, and R95p. R10mm and R95p represent the frequency and threshold of heavy precipitation, respectively. The heavy rainfall is likely to pose serious threats to the ecological environment of the Qilian Mountains, such as soil erosion, flood disaster, etc. Furthermore, the Qilian Mountains are located in the arid area of northwest China, and its ecological landscape is vulnerable to the change in precipitation. Additionally, the ecological landscape in this region has obvious characteristics of altitude gradient. Therefore, it is of practical significance to discuss the future changes in PRCPTOT at various altitudes in the Qilian Mountains. Figure 15 shows the changes in PRCPTOT at various altitudes of the Qilian Mountains at the middle and end of the 21st century. Figure 15a shows that the increment of PRCPTOT is the largest under SSP585 for the altitudes below 3500 m, followed by SSP126 and the smallest under SSP245. However, the increments of PRCPTOT at altitudes above 3500 m will increase with the enhancement of SSP scenarios. The changes in PRCPTOT at various altitudes of the Qilian Mountains at the end of the 21st century are shown in Figure 15b. At the end of the 21st century, the increments of PRCPTOT at all altitudes of the Qilian Mountains are likely to increase with the enhancement of SSP scenarios. In general, the increase of PRCPTOT in high-altitude areas of the Qilian Mountains is the largest. There are a large number of glaciers in the high-altitude area of the Qilian Mountains. This may be because the troposphere is more active under climate warming, and the warm air is easier to form precipitation in the high-altitude glacier area of the Qilian Mountains. At the same time, precipitation is an important factor affecting the change of glacier mass balance. Some studies show that climate warming and increased precipitation will lead to glacier retreat [50,51]. Therefore, under the background of climate warming, the glaciers in the Qilian Mountains will have significant changes by the middle and end of the 21st century.

### 5.3. Biases and Deficiencies in the Projection of Extreme Precipitation

The future projection of extreme precipitation indices over the Qilian Mountains is based on the simulation results of future global climate by CMIP6 models under different SSP scenarios. Some studies show that the applicability of these CMIP6 models in different regions of the world varies to a great extent [32,33]. It is well known that many climate model products have serious deficiencies in the accuracy of precipitation in high-altitude mountainous areas. The three CMIP6 models selected in this study performed relatively well in studying the extreme precipitation indices over the Qilian Mountains, but there is still room for improvement. This is mainly because the simulation of extreme precipitation in mountainous areas needs higher simulation accuracy, and the existing calculation capacity and the applicability of parameterization schemes are difficult to meet the simulation requirements. And each model has its own unique parameterization scheme and mode input, so their simulation results will vary greatly. In this study, three CMIP6 models that have relatively good performance in the precipitation simulation of the Qilian Mountains were selected, but their projection of the future extreme precipitation indices over the Qilian Mountains still has great uncertainty. In addition, this study used an error correction algorithm to correct the output of the CMIP6 model, but the model precipitation correction is based on the observation data of precipitation. However, observation stations in the Qilian Mountains are rare due to the harsh natural conditions in high-altitude mountains. Most of these observation stations are located in the valleys of the Qilian Mountains, and there are elevation errors between them and the grid points of the model. However, there are only limited precipitation gauge stations in the study area, and they are located at lower elevations, which may lead to an underestimation of precipitation. The non-uniform distribution of the observation stations may lead to an inaccurate model evaluation in the area with few stations. These deficiencies in the observation data are likely to affect the assessment of the model in this region [7,19] and may be subjected to potential bias for the correction of the model. The spatial interpolation method based on a small number of observation stations may involve inevitable errors in alpine areas. A previous study [52] suggested that there are differences in the precipitation intensity and frequency of precipitation in low-land to alpine areas. Thus, the interpolation method has always been inadequate in the application of precipitation in alpine areas [53]. Therefore, the improvement of precipitation observation accuracy in the Qilian Mountains is an important basis for future research on precipitation and extreme precipitation.

## 6. Conclusions

In this study, the future projection of the eight extreme precipitation indices over the Qilian Mountains based on three CMIP6 models (CESM2, EC-Earth3, and KACE-1-0-G) was carried out. A bias correction algorithm was used to correct the output of the CMIP6 model, and the extreme precipitation indices of the Qilian Mountains in the historical and future periods based on the corrected CMIP6 model precipitation were calculated. The simulated performance of the corrected CMIP6 model for the extreme precipitation indices in the Qilian Mountains during the historical period as compared to the observations in the 35 meteorological stations was evaluated. At the end of this study, the future spatiotemporal changes in extreme precipitation indices over the Qilian Mountains were analyzed and discussed. The main conclusions are summarized as follows:

(1) On the whole, the corrected CMIP6 models are able to simulate the changes of extreme precipitation indices over the Qilian Mountains in the historical period, but there are still large errors in the simulation of some indices (CDD, R95p, and Rx1day). The CMIP6 models performed well in the simulations of R10mm and PRCPTOT, and the results correlated well with the observed values. CESM2 displays better simulation performance for extreme precipitation indices in the Qilian Mountains as compared to the other two CMIP6 models.
(2) The CDD of the Qilian Mountains is likely to decrease in the middle and end of the 21st century under different SSPs. The remaining seven indices in the Qilian Mountains under the three SSPs will increase in both periods (2041–2060 and 2081–2100). Moreover, the changes in the eight extreme precipitation indices will increase with the enhancement of the SSP scenario. The growth rate of precipitation in the Qilian Mountains during the 21st century under SSP585 is significantly higher than the other two SSP scenarios. The increment in precipitation over the Qilian Mountains mainly comes from the increase in heavy precipitation.
(3) The Qilian Mountains will become wetter in the 21st century, especially in the central and eastern regions. The precipitation intensity and total precipitation of the Qilian Mountains will maintain continuous growth in the middle and end of the 21st century under SSP585 scenario, which is different from SSP126. The western Qilian Mountains are likely to witness the largest increase in precipitation intensity and total precipitation in the middle and end of the 21st century under SSP585.
(4) The inter-annual variation in the drought index (CDD) shows strong volatility both in the historical period and in the future. The remaining seven extreme precipitation indices show a fluctuating upward trend under different SSPs in the 21st century. In addition, the precipitation increment of the Qilian Mountains will increase with the altitude in the middle and end of the 21st century. In the 21st century, the change in precipitation at the high-altitude area of the Qilian Mountains will have an important impact on glacier mass balance.

## Figures and Tables

**Table 1:** Main information on selected GCMs from the CMIP6 used in this study.

Model Name	Institute and Country	Resolution (lat × lon)
CESM2	National Center for Atmospheric Research (NCAR), USA	0.938° × 1.25°
EC-Earth3	EC-Earth-Consortium, Europe	0.703° × 0.703°
KACE-1-0-G	National Institute of Meteorological Sciences/Korea Meteorological Administration (NIMS-KMA), Republic of Korea	1.25° × 1.875°

**Table 2:** Brief description of the eight extreme precipitation indices used in this study.

Index	Descriptive Name	Definition	Units
CDD	Consecutive dry days	Maximum number of consecutive dry days (when precipitation < 1.0 mm)	days
CWD	Consecutive wet days	Maximum annual number of consecutive wet days (when precipitation ≥ 1.0 mm)	days
R10mm	Number of heavy rain days	Number of days when precipitation ≥ 10 mm	days
R95p	Total annual precipitation from heavy rain days	Annual sum of daily precipitation > 95th percentile	mm
Rx1day	Max 1-day precipitation	Maximum 1-day precipitation total	mm
Rx5day	Max 5-day precipitation	Maximum 5-day precipitation total	mm
SDII	Daily precipitation intensity	Annual total precipitation divided by the number of wet days (when total precipitation ≥ 1.0 mm)	mm/d
PRCPTOT	Annual total wet-day precipitation	Sum of daily precipitation ≥ 1.0 mm	mm

**Table 3:** Statistical errors of the eight extreme precipitation indices between the original CMIP6 models and the observed values.

Index	CESM2				EC-Earth3				KACE-1-0-G			
	CC	RMSE	MAE	BIAS	CC	RMSE	MAE	BIAS	CC	RMSE	MAE	BIAS
CDD	0.15	75.81	59.93	−48.08	0.35	70.68	56.74	−37.55	0.26	76.42	60.96	−49.55
CWD	0.28	6.76	5.23	118.44	0.46	5.09	3.51	71.24	0.31	5.16	3.85	79.96
R10mm	0.63	12.34	9.56	149.99	0.64	4.21	3.09	−20.34	0.54	5.00	3.64	−13.13
R95p	0.38	124.06	95.02	183.50	0.42	50.28	37.11	13.58	0.35	53.01	39.21	19.69
Rx1day	0.36	19.16	15.27	53.11	0.36	13.06	9.38	−27.05	0.32	13.05	9.34	−29.25
Rx5day	0.42	41.39	33.68	81.28	0.43	20.32	15.00	−8.26	0.40	19.32	14.40	−12.07
SDII	0.45	1.41	1.10	1.33	0.39	2.08	1.72	−31.75	0.45	2.00	1.65	−31.06
PRCPTOT	0.71	412.36	346.68	148.20	0.83	124.39	90.59	21.61	0.70	166.61	125.08	36.41

**Table 4:** Statistical errors of the eight extreme precipitation indices between the corrected CMIP6 models and the observed values. (* represents the corrected CMIP6 model).

Index	CESM2 *				EC-Earth3 *				KACE-1-0-G *			
	CC	RMSE	MAE	BIAS	CC	RMSE	MAE	BIAS	CC	RMSE	MAE	BIAS
CDD	0.47	54.64	42.61	−1.12	0.48	55.04	42.03	−0.78	0.44	58.20	43.82	3.40
CWD	0.53	2.02	1.43	−0.89	0.45	2.45	1.74	14.57	0.53	2.62	1.89	22.56
R10mm	0.78	3.27	2.37	−0.35	0.74	3.60	2.61	1.20	0.71	3.82	2.78	−0.24
R95p	0.42	46.64	34.37	−2.77	0.38	51.65	37.12	2.55	0.36	51.04	37.15	−2.56
Rx1day	0.45	12.07	8.35	−0.73	0.43	12.59	8.52	−1.87	0.39	12.93	8.99	−2.03
Rx5day	0.55	18.70	13.77	1.51	0.45	21.47	15.51	6.83	0.48	21.45	15.73	8.13
SDII	0.57	1.27	0.95	−0.39	0.57	1.29	0.96	−1.76	0.52	1.38	1.04	−1.80
PRCPTOT	0.88	69.18	51.98	0.30	0.85	80.57	58.91	1.68	0.84	83.99	62.64	0.91

**Table 5:** Average changes of the eight extreme precipitation indices over the Qilian Mountains under different SSPs. The first two columns under each emission scenario are the changes in the middle and end of the 21st century as compared to the base period. The third column under each emission scenario is the change at the end of the twenty-first century as compared to the middle of the twenty-first century.

Index	SSP126			SSP245			SSP585		
	2041–2060	2081–2100	Change	2041–2060	2081–2100	Change	2041–2060	2081–2100	Change
CDD	−4.25	−3.56	0.68	−6.07	−12.83	−6.76	−6.13	−23.02	−16.89
CWD	0.62	0.94	0.32	1.00	1.26	0.26	0.98	1.09	0.11
R10mm	2.09	2.10	0.01	2.23	2.93	0.70	3.14	5.17	2.03
R95p	35.76	29.75	−6.01	37.77	43.93	6.16	57.68	101.44	43.77
Rx1day	6.52	5.17	−1.35	6.10	7.27	1.17	8.08	15.90	7.82
Rx5day	13.77	13.63	−0.14	16.74	16.17	−0.58	19.46	31.47	12.01
SDII	0.94	0.60	−0.34	0.97	0.92	−0.05	1.49	2.12	0.63
PRCPTOT	55.90	58.35	2.45	61.99	81.55	19.56	84.75	151.15	66.40

## Data Availability

Meteorological observation data was provided by the China Meteorological Administration Meteorological Data Center (http://data.cma.cn/, accessed on 10 February 2020). CMIP6 model data is available from https://esgf-node.llnl.gov/search/cmip6/ (accessed on 15 January 2021).

## Appendix A

**Table A1:** Location information of the basic weather stations.

No.	Station Name	Station-ID	Long (dd)	Lat (dd)	Elevation (m)
1	Dunhuang	52418	94.68	40.15	1139.0
2	Guazhou	52424	95.78	40.53	1170.9
3	Yumen	52436	97.03	40.27	1526.0
4	Subei	52515	94.87	39.52	2137.2
5	Jiuquan	52533	98.48	39.77	1477.2
6	Lenghu	52602	93.20	38.45	2770.0
7	Tuole	52633	98.25	38.49	3367.0
8	Sunan	52643	99.62	38.83	2311.8
9	Yeniugou	52645	99.36	38.26	3314.0
10	Zhangye	52652	100.28	39.08	1461.1
11	Minle	52656	100.82	38.43	2281.4
12	Qilian	52657	100.15	38.11	2787.4
13	Yongchang	52674	101.97	38.23	2093.9
14	Wuwei	52679	102.67	37.92	1540.2
15	Da Qaidam	52713	95.21	37.51	3173.2
16	Delingha	52737	97.23	37.22	2981.5
17	Tianjun	52745	99.02	37.30	3417.8
18	Gangca	52754	100.08	37.20	3301.5
19	Menyuan	52765	101.37	37.23	2850.0
20	Gulang	52784	102.90	37.47	2072.4
21	Wushaoling	52787	102.87	37.20	3045.1
22	Jingtai	52797	104.03	37.11	1630.9
23	Chaka	52842	99.05	36.47	3087.6
24	Gonghe	52856	100.37	36.16	2835.0
25	Xining	52866	101.45	36.44	2295.2
26	Ledu	52874	102.38	36.48	1981.9
27	Minhe	52876	102.51	36.19	1813.9
28	Yongdeng	52885	103.26	36.75	2118.8
29	Dingxin	52446	99.52	40.30	1177.4
30	Jinta	52447	98.88	40.00	1270.5
31	Gaotai	52546	99.83	39.37	1332.2
32	Shandan	52661	101.08	38.80	1765.5
33	Minqin	52681	103.08	38.63	1367.5
34	Dulan	52836	98.06	36.18	3189.0
35	Guide	52868	101.26	36.02	2237.1
